# Supplementary material for: Eumelanin Detection in Melanized Focal Changes but Not in Red Focal Changes on Atlantic Salmon (Salmo salar) Fillets
Source: Int J Mol Sci. 2023 Nov 27;24(23):16797. doi: 10.3390/ijms242316797 (PMC10706398; doi:10.3390/ijms242316797)
Supplement: Supplementary file 1 [file ijms-24-16797-s001.zip › ijms-2703025-supplementary.pdf]

Supplementary Tables

Supplemental Table S1

Average values of spectrophotometric and chemical degradation analyses of control, MFC (BS-small, BS-medium, BS-large) and RFC (RS-small, RS-medium, RS-large) in Materials #1. The number of samples in each group = 3.

	A500 x 1000 (1/mg)	A650/A500 x 100	PTCA (ng/mg)	PDCA (ng/mg)	4-AHP (ng/mg)	3-AHP (ng/mg)	HI-DOPA (ng/mg)	PB-5SCD (ng/mg)	4-AHP/3-AHP
Control	6.5	2.6	0.72	0.23	0.26	0.13	4.7	2.3	2.0
BS-small	7.3	6.8	3.4	0.32	0.15	0.28	2.4	2.6	0.54
BS-medium	9.7	18.2	7.0	0.42	0.12	0.30	4.5	2.1	0.40
BS-large	14.7	22.0	19.4	0.95	0.13	0.53	4.9	2.2	0.25
RS-small	14.8	6.8	2.1	0.24	0.47	0.57	10.3	4.3	0.83
RS - medium	19.6	5.3	1.6	0.27	0.66	1.0	15.3	5.2	0.66
RS - Large	20.8	5.0	0.7	0.23	0.71	0.85	17.0	5.1	0.83

Supplemental Table S2

Average values of spectrophotometric and chemical degradation analyses of control (n = 2), MFC (BS-small, n = 3; BS-medium, n = 17; BS-large, n = 7) and RFC (n = 3) in Materials #2.

	A500 x 1000 (1/mg)	A650/A500 x 100	PTCA (ng/mg)	PDCA (ng/mg)	4-AHP (ng/mg)	3-AHP (ng/mg)	HI-DOPA (ng/mg)	4-AHP/3-AHP
Control (n = 2)	7.5	6.7	0.33	0.30	0.13	0.74	15.3	0.18
BS - small (n = 3)	8.3	9.0	2.5	0.45	0.11	0.68	10.1	0.16
BS - medium (n = 17)	9.2	15.8	7.8	0.60	0.19	0.90	11.0	0.21
BS - Large (n= 7)	11.6	18.6	13.7	0.70	0.14	0.47	12.5	0.30
RFC (n = 3)	24.6	9.9	0.35	0.55	0.61	1.2	18.8	0.51

Supplementary figures

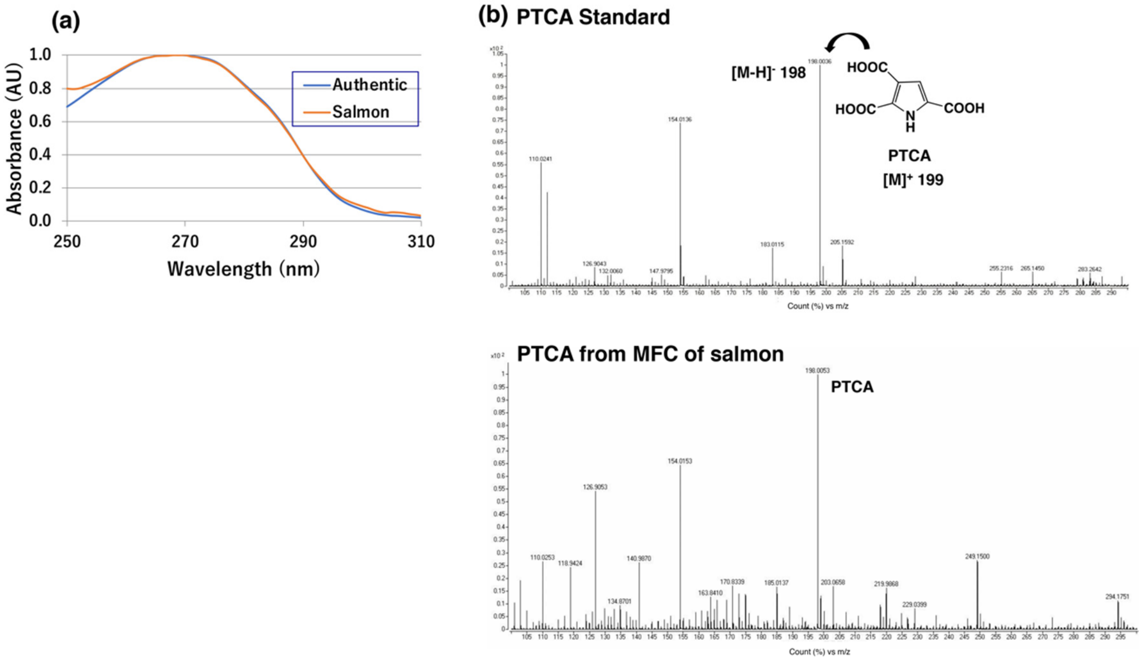

Supplemental Figure S1. The identification of PTCA was obtained by isolating PTCA in a preparative scale AHPO in MFC. The UV-VIS (Suppl. Fig. S1a) and Mass spectra (Suppl. Fig. S1b) were confirmed by the comparison of authentic PTCA samples and PTCA isolated from MFC of salmon.

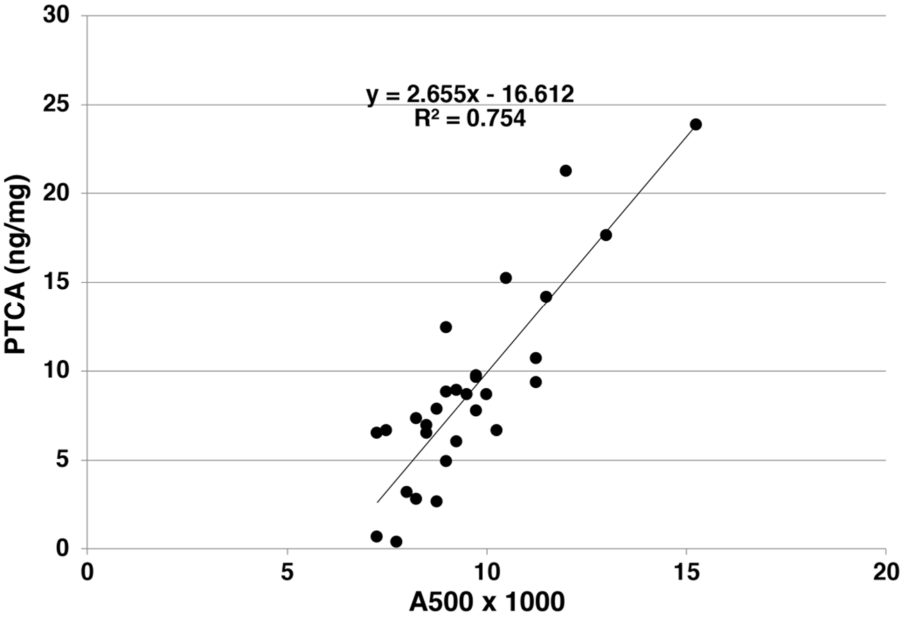

Supplemental Figure S2. The correlation of PTCA with A500 values. The PTCA values correlated well (R^2 = 0.754) with the A500 values with a background value of 0.006/mg. This shows that MFC is mostly due to EM.
